# Peptidyl prolyl *cis*/*trans* isomerase activity on the cell surface correlates with extracellular matrix development

**DOI:** 10.1038/s42003-019-0315-8

**Published:** 2019-02-11

**Authors:** Weilin Lin, Malte Bonin, Annett Boden, Robert Wieduwild, Priyanka Murawala, Martin Wermke, Helena Andrade, Martin Bornhäuser, Yixin Zhang

**Affiliations:** 1 B CUBE Center for Molecular Bioengineering, Technische Universität Dresden, Dresden, 01307 Germany; 2 Medizinische Klinik und Poliklinik I, Universitätsklinikum Carl Gustav Carus, Dresden, 01307 Germany; 3 German Consortium for Translational Cancer Research (DKTK), partner site Dresden, Dresden, Germany; 4 German Cancer Research Center (DKFZ), Im Neuenheimer Feld 280, Heidelberg, 69120 Germany

## Abstract

Interactions with the extracellular matrix (ECM) dictate cell fates. However, the complexity of dense ECM network and cell-surface molecules prevent the study of their dynamic interaction at the molecular level on living cells. Here, we focus on peptidyl prolyl *cis/trans* isomerases (PPIases) to dissect prolyl isomerization from other dynamic events. We reveal the contribution of PPIase on the mechanical properties of various ECM materials and on the dynamic cell–ECM interaction. To avoid complications associated with the existing spectroscopy-based methods such as light scattering, an assay was developed for detecting PPIase activity on living cell surface. This assay allows us to correlate PPIase activity with ECM development, and with the physiological and pathological states of the cells, including the functional properties of cancer cells and immune effector cells.

## Abstract (web summary)

Weilin Lin et al. report a new assay for measuring the activity of peptidyl prolyl *cis/trans* isomerase (PPIase) at the cell surface using ultra performance liquid chromatography. They find that PPIase activity correlates with development and functional properties of the extracellular matrix in primary healthy and leukemic cells.

## Introduction

The dynamics of polypeptide chains in complex biological systems are temporospatially controlled. They can be affected not only by various post-translational modifications (e.g., phosphorylation, acetylation, and glycosylation), but also by the catalytic activity of foldases. Among the foldases, peptidyl prolyl *cis*/*trans* isomerases (PPIases) catalyze the isomerization between the *cis* and *trans* forms of peptide bonds, which are associated with the polypeptide conformation by the 180° rotation about the prolyl bond. By catalyzing protein conformational changes, PPIases regulate the molecular interaction and enzymatic reaction, and could act as the molecular timer in various physiological and pathological processes^1,2^.

There are three families of PPIases^3^. Cyclophilins (Cyps) and FK506 binding proteins (FKBPs) are receptors for the immunosuppressive drugs cyclosporin A (CsA) and FK506, respectively^4^, while the parvulin family, best known for its member Pin1, has been found to be involved in cellular cycles, Alzheimer’s disease, and cancer^5,6^. The catalytic effects of PPIases on the folding, dynamics, and function of different proteins have been intensely studied. PPIases bind to extracellular matrix (ECM) proteins, for eg, collagen^7^ and hensin^8^, and catalyze their folding. However, whether PPIases directly regulate the structural dynamics of the dense polymer network of ECM and the complex cell surface proteins, thus affecting their interaction, has not been investigated so far to our knowledge.

The ECM undergoes continuous remodeling, orchestrated through its synthesis and secretion by cells as well as through the degradation by specific enzymes, for e.g., metalloproteinases. The dynamics can affect their biochemical and mechanophysical properties and can further dictate tissue-specific cell behavior^9^. While the effect of catalyzed folding on ECM properties remains largely elusive, an assay for the direct detection of PPIase activity on living cells is still missing. Herein, we have developed assays to reveal the presence and activity of PPIase associated with ECM and different cell types. A video abstract of this study is presented in Supplementary Movie 1.

## Results

### Effect of CypA on the rheological properties of ECM mimics

Studying ECM or cell surface proteins by staining-based techniques (e.g., immunofluorescence or western blot) can only measure the individual protein semi-quantitatively. It neglects structural dynamics and functional regulation, such as inhibition or limited diffusion upon binding to the matrix. To directly investigate the effect of PPIase on ECM dynamics, we tested the influence of PPIases on the gelation and stiffness of various ECM biomaterials using a rheometer. The storage modulus from the rheometer depends on the elastic component of a viscoelastic material and reflects the sample’s stiffness.

**Fig. 1:**
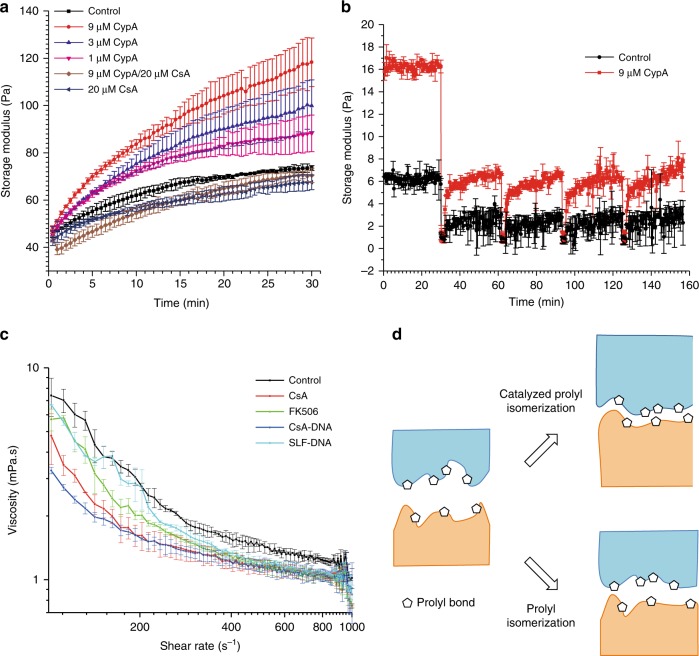
Effect of PPIase on ECM dynamics and dynamics interaction of cell–ECM. Enhanced stiffness (storage modular) of fibrin hydrogel (**a**) by cyclophilin. The effects can be fully inhibited by cyclophilin inhibitor CsA. **b** In a step-strain assay, the self-healing of collagen hydrogel is enhanced by cyclophilin after physical damage. **c** Viscosity measurements of Jurkat T lymphocyte in plasma protein fibrinogen solution with or without PPIase inhibitor (CsA, FK506, CsA-DNA, and SLF-DNA) treatment. Through inhibiting the PPIase activity, the cells become more “slippery” upon interacting with the plasma protein and exhibit reduced viscosity. **d** Hypothesis of the catalysis effect by PPIases. PPIases can accelerate structural changes under non-equilibrium conditions and thus contribute to various dynamic processes involving biogenic material interfaces. CsA, cyclosporin; ECM, extracellular matrix; PPIases, peptidyl prolyl *cis*/*trans* isomerases

The gelation of fibrin is initiated by fibrinogen proteolysis with thrombin. In the presence of 1 µM cyclophilin A (CypA), the storage modulus was remarkably enhanced (Fig. 1a). Increasing CypA concentration further increases the hydrogel stiffness, and the enhanced effect can be fully inhibited by CsA. We performed the measurement with CypA-inactive mutant R55A. As compared to the wild-type CypA, the effect of CypA mutant on fibrin gelation is remarkably reduced (Supplementary Fig. 1). As the rearrangement of ECM network could be associated with a large amount of prolyl isomerization, it is unlikely that the effect involves only a specific peptidyl prolyl bond. Unlike the classical spectroscopy-based PPIase activity assays, the rheology-based method provides a macroscopic measurement of the effect of catalyzed peptidyl prolyl *cis/trans* isomerization. The effect of CypA on the gelation of biomaterials was further confirmed by the pH-induced and temperature-induced gelation of collagen and the temperature-induced gelation of Matrigel, respectively (Supplementary Fig. 2).

Another important feature of the dynamic ECMs is their self-healing property, which can be tested using a step-strain rheological measurement. A strong strain is applied to liquify the materials, and the recovery of storage modulus is followed (self-healing) (Fig. 1b). Collagen hydrogel can only partially recover its storage modulus after the first cycle. The rate and extent of recovery were unchanged over the following cycles. In the presence of CypA, not only the stiffness but also the extent of recovery in the self-healing process can be remarkably enhanced.

### Tuning the interaction between cells and ECM by PPIases

Since PPIases can catalyze the dynamic rearrangement of the dense ECM polymer network, we speculated that PPIases could also affect the dynamic interaction between cells and the ECM. Probing the influence of transient prolyl isomerization on cell–ECM interaction is difficult. Studying cell adhesion by the treatment of PPIase inhibitors cannot distinguish extracellular and intracellular factors as well as receptor-mediated signaling due to the long assay time. In order to reflect the highly dynamic process, we measured the viscosity of plasma protein solution with cells. Blood is a shear-thinning non-Newtonian fluid, and its viscosity is largely affected by the interaction between cells and plasma proteins, for example, the coagulation process. We investigated whether the catalyzed isomerization could also influence the interaction between blood cells and plasma proteins, thus affecting the viscosity. We performed viscosity/shear rate measurements using a mixture of Jurkat T lymphocyte and the plasma protein fibrinogen. The measurements are highly reproducible in the range of 100–1000 s^−1^ of shear rate, which is relevant for body bloody flow^10^. While neither CsA nor FK506 has an effect either on cells in phosphate-buffered saline (PBS) or fibrinogen solution (Supplementary Fig. 3), both compounds can reduce the viscosity of cell/fibrinogen mixture (Fig. 1c). To further exclude the possible effect of drug treatment on intracellular pathways, we synthesized CsA–DNA and SLF–DNA conjugates (SLF, Synthetic Ligand of FKBP, is an FK506 derivative and FKBP inhibitor), which inhibit Cyps and FKBPs, respectively, and do not enter into cells (Supplementary table 1, Supplementary Figs. 4 and 5). Both DNA conjugates exhibited similar effects like CsA and FK506. Through inhibiting the catalyzed prolyl isomerization, thus suppressing the interaction kinetics between cells and fibrinogen, the cells become more “slippery” upon interacting with the plasma protein and exhibit reduced viscosity.

### The hypothesis of prolyl bond dynamics at cell–ECM interface

We postulate that the catalyzed *cis/trans* isomerization can contribute to ECM dynamics associated with the adaptive behavior of cells (Fig. 1d). If the encounter of cells to a specific microenvironment is limited to a short period of time (e.g., circulation of lymphocytes in the blood stream), the interaction is controlled not only thermodynamically but also kinetically. The rate of dynamic rearrangement of ECM and cell surface molecules would influence the adhesion through molecular recognition and binding. Cyp has been previously shown to affect the adhesion of T lymphocytes as chemokine, although the effect was attributed to the CD147-medieated intracellular signaling^11^. However, as CypA and cyclophilin B (CypB) have been primarily discovered as PPIases, they could also modulate the pathophysiological state of cells through accelerating the prolyl bond rotation, thus affecting protein dynamics. To answer this question, a direct analysis of PPIase activity of living cells is still missing. Most foldase assays are time-dependent measurements. To avoid complications associated with the diffusion of reagents through cell membrane and difficulties to establish a transient non-equilibrium condition in cells, in this work we aim to measure PPIase activity on the living cell surfaces (PACS).

### High-throughput assay for PPIase activity measurements

**Fig. 2:**
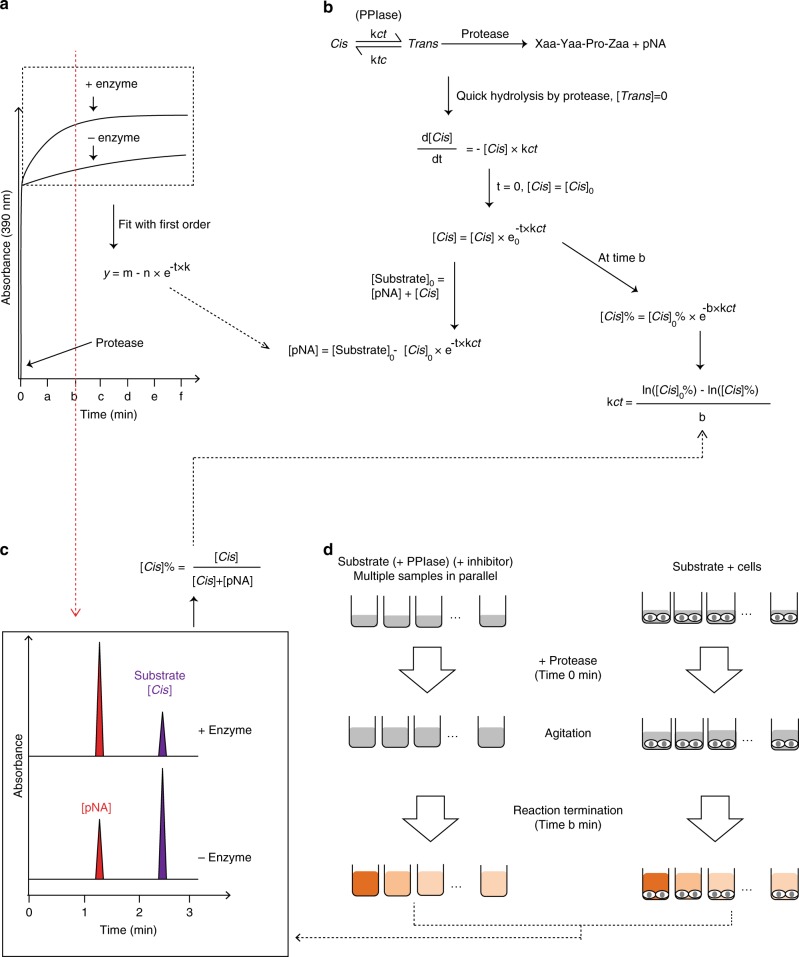
Principle of PPIases assay. **a** PPIase activity measurement based on the kinetic monitoring of pNA production using UV–Vis spectrometer. The curves (inside rectangle) were first-order fitted. The *k* value is equal to the *k*_*ct*_ value in (**b**). **b** Calculation of PPIase activity resulting from protease-coupled assays. *k*_*ct*_ and *k*_*tc*_ are the rates of *cis* to *trans* and *trans* to *cis* isomerizations, respectively. In a protease-coupled assay, the activity of PPIase is *k*_ct+enzyme_*-k*_ct_. [*Cis*]_0_ is the initial *cis* concentration after proteolysis of all the *trans* isomers. **c** PPIase activity measurement based on liquid chromatography. When proteolysis is terminated at time *b* instantly, the [*Cis*]% can be determined precisely. **d** Measurement of PPIase activity in solution (left panel) or with cells (right panel). After incubation of the substrate in the presence or absence of PPIase, or cells, and/or inhibitor, the reaction was triggered by adding protease with agitation, stopped at time *b*, and the mixtures were analyzed by liquid chromatography. PPIase, peptidyl prolyl *cis*/*trans* isomerase

**Fig. 3:**
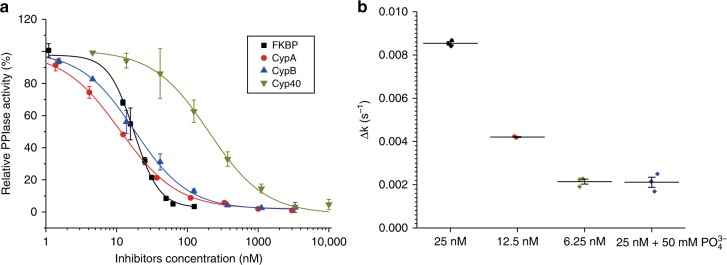
Parallel activity and inhibition measurements. **a** Inhibition of FKBP12 by rapamycin (black square) and inhibition of CypA (red cycle), CypB (blue upward pointing triangle), and Cyp40 (dark yellow downward-pointing triangle) by CsA. PPIase activities are shown as the mean of triplicates ± SD and expressed as percentages of enzyme activity relative to an inhibitor-free control. The *K*_i_ values were 1.5 ± 1.2 nM for FKBP/rapamycin, 8.6 ± 1.2 nM for CypA/CsA, 12.9 ± 1.3 nM for CypB/CsA, and 176.8 ± 27.6 nM for Cyp40/CsA. The resulting IC_50_ values are in good agreement with the previously reported values using standard PPIase assays. **b** Concentration-dependent Pin1 activity. The addition of PO_4_^3-^ resulted in Pin1 inhibition. CsA, cyclosporin A; CypA, cyclophilin A; CypB, cyclophilin B; Cyp40, cyclophilin 40; PPIase, peptidyl prolyl *cis*/*trans* isomerase

The foldase activity of PPIases was first discovered using a protease-coupled assay, which takes the advantage of proteases (e.g., α-chymotrypsin)-cleaving substrates when the prolyl bonds are in *trans* conformation^12^. The further developments in PPIase assays^3^ have been limited to spectroscopic detection, which must be performed in transparent solutions, and can be affected by any artifacts causing light absorption or scattering. We developed a liquid chromatography-based high-throughput PPIase activity assay. When the protease-coupled process is terminated at a certain time point, the substrate and product can be separated and quantified using liquid chromatography, allowing for the calculation of the isomerization rate (Fig. 2). We applied this method to measure the inhibition constants of CsA to CypA, CypB, and cyclophilin 40 (Cyp40), as well as the inhibition constant of rapamycin to FKBP12 (Supplementary Figs. 6–8) The resulting IC_50_ values are in good agreement with the previously reported values using standard PPIase assays (Fig. 3a)^13,14^. Using the same protocol, the PPIase activity of Pin1 could also be measured with Suc-Ala-Asp-Pro-Phe-pNA as the substrate and nagarse as protease. In good agreement with the previous report, inorganic phosphate (50 mM) inhibited Pin1 activity^15^(Fig. 3b). This method avoids the complications associated with the existing spectroscopy-based methods, such as light scattering by cells. For example, we can measure the PPIase activity of Cyp immobilized on silica beads (Supplementary Fig. 9). It is important to note that the assay is high throughput as multiple measurements can be performed in parallel.

### PPIase activity measurement on cell surface

**Fig. 4:**
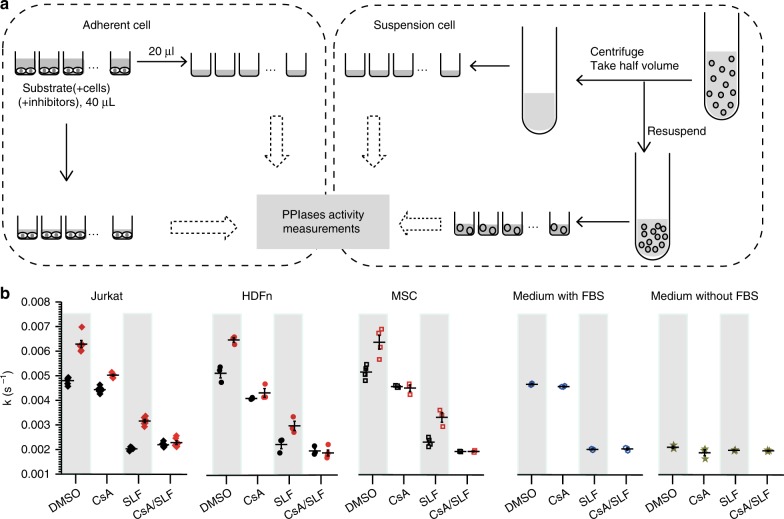
PACS assay. **a** Scheme of activity measurements for adherent cells (left panel) and suspension cells (right panel). Adherent cells were seeded and incubated with the substrate in the presence or absence of inhibitor. Half of the volume was taken to measure the activity in the supernatant and the other half was used to measure the activity with cells. The harvested suspension cells were incubated with the substrate. After centrifugation, half of the volume was taken to measure the activity, and the other half (cell pellet with supernatant) was re-suspended and used for activity measurements. **b** PACS of Jurkat cells, HDFn, and MSCs. Black dots: in supernatant; red dots: with cells; blue dots: CO_2_-independent medium with FBS control; dark yellow dots: CO_2_-independent medium without FBS control. PPIase activities resulted mainly from Cyps (inhibited by CsA, but not SLF). FBS, fetal bovine serum; HDFn, human dermal fibroblasts isolated from neonatal foreskin; MSCs, mesenchymal stromal cells

To investigate PACS, we need to distinguish between different families of PPIase. Cyp and FKBP, but not parvulin, can be selectively inhibited by CsA and FK506, respectively, hence enabling us to distinguish these three protein families pharmacologically. PACS must also be distinguished from that in culture medium. Adhesive cells such as fibroblasts (human dermal fibroblasts isolated from neonatal foreskin (HDFn)) or mesenchymal stromal cells (MSCs) were washed by simple pipetting and pooling. Washing of suspension cells such as T cells was performed by centrifugation. After the first washing step, no change in PPIase activity in supernatant could be detected through the following washing steps. To minimize the potential damage to cells, one washing step was used in the PACS measurement for suspension cells (Supplementary Fig. 10). After washing, the assays were performed as shown in Fig. 4a, to compare the PPIase activity in the presence or absence of cells. There was residual PPIase activity in the supernatant, which was close to the background activity in culture media, and could be completely inhibited by FKBP inhibitor SLF, but not by CsA (Fig. 4b, first right). Interestingly, PACS could be attributed to Cyps, as adding CsA abolished the difference between the samples with cells and the supernatants without cells. Adding SLF further reduced the isomerase activity in samples both with and without cells due to the FKBP in the fetal bovine serum (FBS)-containing medium. After the treatment of CsA/SLF, these was no difference in the substrate *cis*/*trans* isomerization rate in all the samples. Therefore, neither the cells nor the FBS in the culture medium interferes with the protease-coupled PPIase assay (Fig. 4b).

### PACS and ECM development of adherent cells

**Fig. 5:**
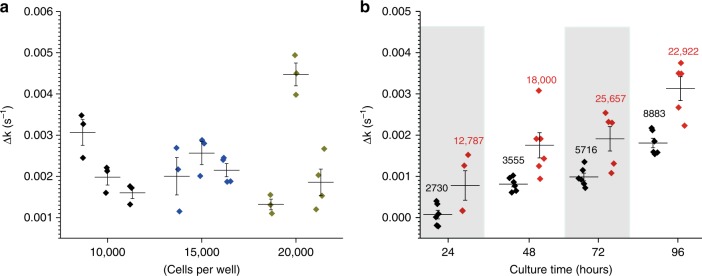
The accumulation of PPIases associated with the development of ECM. **a** PACS on HDFn with differing cell numbers seeded. Three independent experiments were performed with three different cell numbers. No correlation was observed between fibroblast cell number and itsPPIase activity. **b** PACS on HDFn cells with different culture times. Two thousand (black dots) and 10,000 (red dots) cells/well were seeded and cultured, and the PPIase activity was measured after 24, 48, 72, and 96 h. The inserted numbers represent the actual cell numbers/well after culturing. The PACS showed a correlation with culture time. Δ*k* (*y*-axis) was obtained after subtraction of PPIase activity with the cells from the activity in the corresponding supernatants. HDFn, human dermal fibroblasts isolated from neonatal foreskin; PPIase, peptidyl prolyl *cis*/*trans* isomerase

**Fig. 6:**
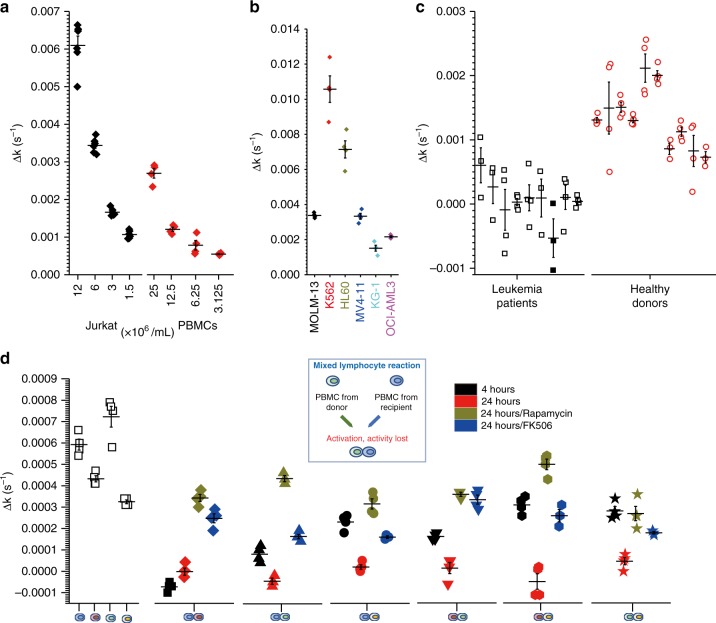
The connection of PACS with the physiopathological state of the cells. **a** Cell number-dependent PACS on Jurkat T cells and PBMCs. **b** PACS on six leukemia cell lines (cell density = 1.25 × 10^7^ cells/mL). **c** PPIase activity on nine leukemia patient samples (nonfilled symbols, cell density = 1.25 × 10^7^ cells/mL; filled symbols, cell density = 5 × 10^7^ cells/mL) and ten healthy donors (the PACS of 1.25 × 10^7^ cells/mL is a corrected value since some donors have less cell density). **d** PACS in a two-way mixed lymphocyte reaction (cell density = 0.8 × 10^7^ cells/mL). After 4 h, a remarkable decrease in activity was observed. After 24 h, diminished activity was detected in all combinations. Rapamycin and FK506 can recover the PACS. Δ*k* (*y*-axis) was obtained by subtracting PPIase activity with the cells from the activity in the corresponding supernatants. PBMCs, peripheral blood mononuclear cells; PPIase, peptidyl prolyl *cis*/*trans* isomerase

Given that the catalytic effect is caused by enzymes bound to cell membranes and ECM, the resulting activity could be affected by many factors, including cell number, size and morphology, and, probably more importantly, the development of ECM. Interestingly, the PPIase activity on fibroblasts is related not only to the cell number (Fig. 5a). Culturing newly seeded fibroblasts resulted in changes in PPIase activity over time (Fig. 5b). Whereas the newly seeded cells exhibited minor activity, a remarkable increase of activity was observed over the following 4 days, indicating the accumulation of PPIase associated with the development of ECM. Unlike adhesive cells, the PPIase activities on Jurkat T cells and peripheral blood mononuclear cells (PBMCs) in suspension have shown a linear correlation with cell number (Fig. 6a).

### PACS and pathophysiological state of lymphocytes

Much evidence is emerging about the fact that the interaction with a hematopoietic microenvironment can affect the apoptosis, differentiation, and proliferation of leukemia cells^16^. We wonder whether oncogenesis also involves a change in PACS and whether there is a difference between primary patient samples and immortalized leukemia cell lines. We measured the PPIase activity of six leukemia cell lines and nine primary samples from leukemia patients, and compared them with ten PBMC samples from healthy donors (Fig. 6b, c). The primary leukemia samples were derived from patients at the initial diagnosis before chemotherapy or antibiotic treatment was initiated. Interestingly, all nine primary samples exhibited diminished PPIase activity. In one sample, no activity could be detected even when fourfold more cells were used (Fig. 6c; squares in black). Astonishingly, in contrast to primary samples, four of six leukemia cell lines and Jurkat T cells exhibited enhanced PACS, as compared to PBMCs. The difference in PACS of various types of lymphocytes indicates the involvement of regulated ECM dynamics in the gradual adaption of cells to the pathogenic or culturing environments.

### PACS and T-cell activation

To investigate whether the changes in PACS can be directly induced on lymphocytes in response to an immunological stimulation, we performed a two-way mixed lymphocyte reaction. PACS were measured with four PBMC samples from healthy donors or with all six different combinations. Interestingly, 4h after mixing the cells, a remarkable decrease in activity was observed. After 24 h, diminished activity was detected in all combinations (Fig. 6d). For two combinations, no trace of activity could be detected, resembling the results from some primary samples of leukemia cells. Stimulation of immune cells does not only cause cascades of intracellular signaling but also induces a dramatic change in ECM dynamics, which could be essential for the interaction between cells and their hematopoietic microenvironment.

To investigate whether the change is also associated with immune signaling and can be inhibited pharmacologically, we treated the PBMCs with immunosuppressive drugs, such as rapamycin or FK506. Although rapamycin and FK506 inhibit PPIase FKBP, they can be used in this assay because PACS has been attributed to Cyps. As shown in Fig. 6d, both rapamycin and FK506 can recover PACS. Mixed lymphocyte reaction is a routine assay used to measure the safety of implantable materials and the effect of immunosuppressive treatments. The PACS assay could become an alternative test, within a relatively short period of time, avoiding the use of biochemical reagents such as antibodies.

## Discussion

**Fig. 7:**
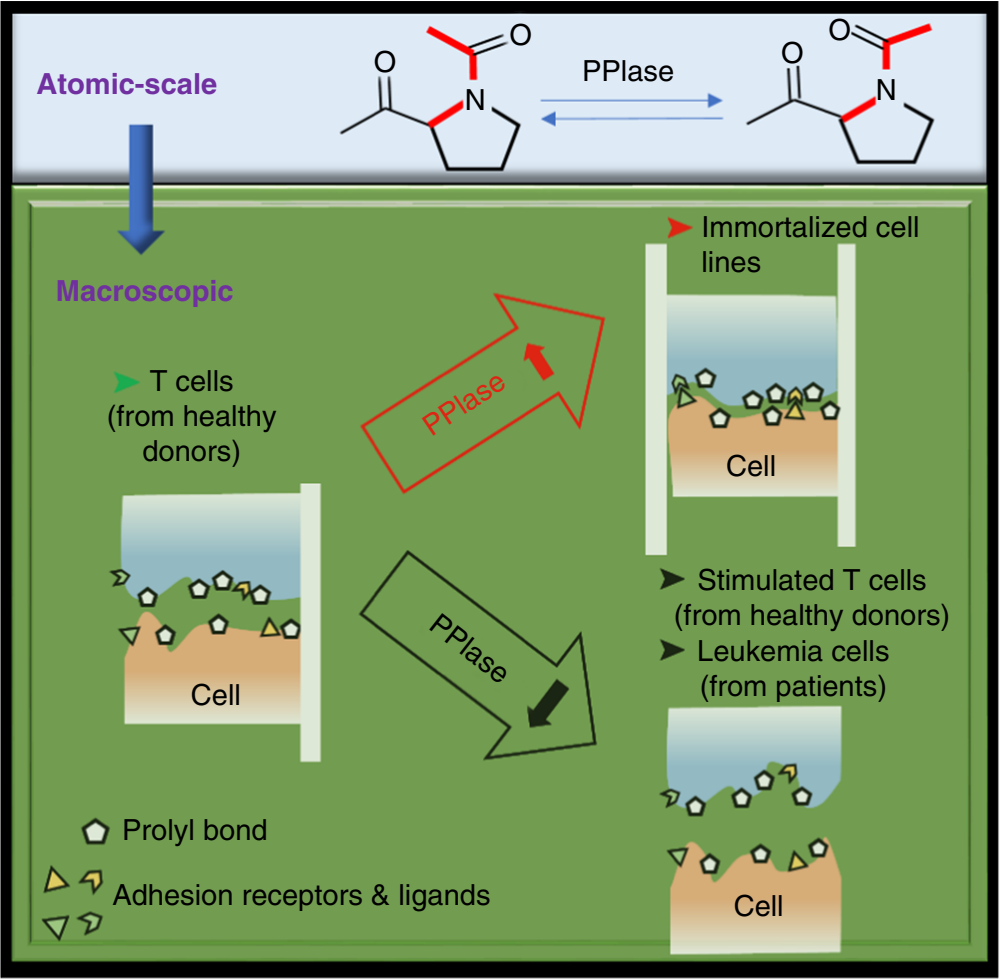
Cells are constantly subjected to non-equilibrium conditions. Catalyzed prolyl bond *cis*/*trans* isomerization functions (atomic-scale) as a molecular timer to regulate their interaction with extracellular matrices and plasma proteins (macroscopic)

The development of new methods to measure the parameters of cells not detectable by existing technologies could open new doors to study cell biology in different ways, for example, cellular mechanics^17^, viscosity^18^, crowding^19^, or fundamental biochemical processes as simple as peptide bond *cis/trans* isomerization^20^. The chromatography-based PPIase assay described in this report avoids the complications associated with spectroscopy-based kinetic measurements and allows many measurements to be performed in parallel in a high-throughput fashion. More importantly, we can measure PPIase activity on cell surface. Astonishingly, primary leukemia patient samples and immortalized cell lines showed changes in PACS in opposite directions. Interestingly, the down-regulation of PACS seems intrinsically coupled to T-cell activation, while all combinations of mixed lymphocyte reactions resulted in reduced PACS. Moreover, although rapamycin and FK506 suppress immune system through completely different mechanisms (calcineurin–NFAT and mTOR pathways, respectively)^21,22^, both the immunosuppressive drug treatments can recover the PACS. The allo-immune response involves various effector and target cells as well as many different signaling molecules and pathways. The diminished PPIase activity could reflect a general and intrinsic change in cell-surface property in response to the activation and reduce their kinetically controlled interaction with ECM proteins (Fig. 7). Other cellular processes, such as oncogenesis and immortalization of primary cells, also involve not only intracellular signaling cascades but also changes in ECM dynamics, and the fates of cells are regulated through their interaction with their environment.

Mass spectrometry or antibody-based detection method can be used to measure the amount of PPIases. However, their quantities do not necessarily correlate with catalytic activity. We consider that the observed activity on certain types of cells is a sum of many factors, including the ECM network properties, the restricted diffusion of different PPIases and their substrates, the competition between the peptide substrate and the endogenous proteins to interact with the enzymes, and the concentrations of different PPIases.

As the current PPIase assays are all related to certain substrates, they cannot detect enzymatic activity toward unknown substrates. By developing new substrates, PACS associated with FKBP might be revealed, as the inhibition of FKBP has shown an effect in the viscosity/shear-thinning assay. The rheology-based PPIase measurements represent a new avenue to study PPIase, providing macroscopic analyses (viscosity and storage modulus) using cells and complex ECM proteins, instead of focusing on short peptide substrates. For example, the changes in ECM dynamics catalyzed by PPIases could be directly assayed using the rheology measurement of the three most widely used ECM mimicries. Because myriads of prolyl bonds in different sequences contribute to the matrix dynamics, it can be used as an alternative method to discover new classes of foldases.

The PPIases, especially Cyps, are highly abundant proteins, while Cyps account for about 0.4% of the dry mass of cellular proteins^23^. Together with their high catalytic activity^24^, these evidences suggest that PPIases possess a broad spectrum of substrates. Investigation into the functions of PPIase on cells gives us a unique opportunity to dissect the prolyl bond rotation from myriads of other structural movements and provides insights into the effect of polypeptide chain dynamics in a biologically relevant context. Furthermore, the PACS assay could provide a simple and alternative method to monitor the pathophysiological states of cells, such as activation of immunity or oncogenesis. Additionally, while the structure and mechanics of biomaterials are important factors to recapitulate cell-specific ECM environments, PPIase processing offers a new tool for biomaterial engineering.

## Methods

### Protein purification

CypA plasmid (pQE-T7-1/CypA), CypB plasmid (pQE-T7-1/CypB), His-Cyp40 plasmid (pQE-T7-1/Cyp40), and His-Pin1 plasmid (pQE-T7-1/Pin1) were purchased from Qiagen (Supplementary Table 2). The plasmid of FKBP was a gift from Gunter Fischer.

Recombinant CypA, CypB, Cyp40, and Pin1 were expressed in *Escherichia coli* (*E. coli*) strain BL21 (DE3) purchased from Stratagene (La Jolla, CA, USA). Pre-culture: single colonies of *E. coli* from the agar plate were picked and inoculated into 20 mL of lysogeny broth medium containing kanamycin (50 µg mL^−1^). The cultures were grown overnight at 37 °C with vigorous shaking. Culture: 15 mL of the overnight pre cultures was diluted in 1 L of pre-warmed medium (2 × YT, 50 µg mL^−1^ kanamycin) and incubated at 37 °C (around 3 h) with vigorous shaking until OD_600_ = 0.8~1. Expression: the expression of target recombinant protein was induced by adding isopropyl β-D-1-thiogalactopyranoside to a final concentration of 300 µM and again incubating overnight at 25 °C (200 rpm shaking). Cells were harvested by centrifuging at 8000 rpm for 20 min and the supernatant was discarded.

For purifying the protein, cells were resuspended in 50 mL buffer A (35 mM hydroxyethyl-piperazineethane-sulfonic acid buffer (HEPES), 500 mM NaCl, 40 mM imidazole, 1 mM dithiothreitol (DTT), and 1 mM phenylmethylsulfonyl fluoride). DTT and phenylmethylsulfonyl fluoride were added freshly in buffer A. Subsequently, the cell suspension was passed through a French press (EmulsiFlex-C3; AVESTIN) at 4 °C under 1000 psi for three times. The lysate was centrifuged at 30,000 rpm at 4 °C for 1 h (Beckmann LE-80K ultracentrifuge; Beckmann, PaloAlto, CA, USA; rotor SW 32Ti). The supernatant was collected and loaded on a Histrap HP column (GE Healthcare GmbH, Munich, Germany) at 4 mL/min. The column was washed with 100 mL of buffer A and the target protein was eluted using a linear gradient up to 100% buffer B (35 mM HEPES, 500 mM NaCl, and 500 mM imidazole) over 30 min. The fractions showing PPIase activity were pooled and concentrated to 500 µL and injected on a HILOAD 16/60 Superdex 75PG (GE Healthcare GmbH) running under buffer C (35 mM HEPES, 200 mM NaCl, and 5% glycerol, pH 7.4). The fractions with PPIase activity were collected, concentrated, and analyzed by Waters ACQUITY UPLC system equipped with an ACQUITY TQ Detector (Milford, Massachusetts, USA) and an analytical C8 column (Waters ACQUTIY UPLC BEH C8; bead size 1.7 µm, 2.1 × 100 mm).

Expression and purification of recombinant human FKBP were performed as described earlier^25^.

### Synthesis of chemical compounds

CsA* (named as CsAaa), Fmoc-CsA-COOH, and SLF-COOH were synthesized according to a previous description with some modifications^26^.

*2-(((Benzyloxy)carbonyl)amino)pent-4-enoic acid (CbzAG)*: DL-2-allylglycine (4 mM) and sodium bicarbonate (8 mM) were dissolved in 30 mL ice cold H_2_O under stirring conditions. To the stirred solution, benzyl chloroformate (6 mM in 20 mL anhydrous tetrahydrofuran) was added drop wise. The resulting mixture was stirred at 0 °C for 1 h and allowed to warm to room temperature overnight. The pH of the reaction mixture was adjusted to 1 with 10% HCl, and the mixture was dried by a rotary evaporator. Five milliliters of acetonitrile was added to the powder. The mixture was filtered through a 0.22 µm polyvinylidene difluoride filter and purified by high-performance liquid chromatography (HPLC).

CsA*: CsA (1 mM), CbzAG (3 mM), and catalyst (Hoveyda-Grubbs Catalyst 2nd Generation) (0.15 mM) were dissolved in 4 mL of dry dichloromethane (DCM) in a 10 mL round flask. The solution was refluxed at 45 °C for 24 h under N_2_. The solvent was evaporated by a rotary evaporator. The dark brown product was dissolved in 50 mL EtOH with 1 mL H_2_O in a 250 mL round flask. Then 200 mg palladium on carbon (Pd/C) was added. The solvent was stirred under H_2_ for 2 h, in the absence of light. The mixture was filtered through a 0.22 µm polyvinylidene difluoride filter to remove the Pd/C, and then 450 mL MilliQ H_2_O was added. The mixture was filtered through the 0.22 µm cellulose filter again to remove the non-reacted CsA. The solvent was lyophilized. The pellet was dissolved in acetonitrile and purified by HPLC and analyzed by liquid chromatography–mass spectrometry (LC/MS). MW= 1277.7; found [M + H]^+^ = 1278.87.

*Fmoc-CsA-COOH*: CsA* (0.326 mM, dissolved in 1.5 mL dimethylformamide (DMF)) and Fmoc-Cl (0.489 mM, dissolved in 1 mL DMF) were stirred in a 10 mL round flask at 0 °C. 435 μl of DIPEA (1.5 M in DMF) was added to the mixture drop wise under stirring. The resulting mixture was stirred at 0 °C for 1 h and allowed to warm to room temperature overnight. The solvent was evaporated and the pellet was dissolved in 5 mL acetonitrile, purified by HPLC, and analyzed by LC/MS. MW= 1499.9; found [M + H]^+^ = 1500.27.

*SLF-COOH*: SLF (12 mg) and succinic anhydride (7 mg) were dissolved in 100 μl DMF, then 28.7 μl of triethylamine was added, and the mixture was stirred overnight. The product from the reaction mixture was purified by HPLC and analyzed by LC/MS. MW= 624.7; found [M + H]^+^ = 625.3.

*Cs9TAMRA*: Twelve microliters of 100 mM Carboxytetramethylrhodamine, N-hydroxysuccinimide ester (TAMRA NHS) in DMF was added to 10 µL of 100 mM Cs9 (gift from Gunter Fischer)^27^ in DMF, and then 10 µL of 300 mM N,N-Diisopropylethylamine (DIPEA) in DMF was added. The mixture was shaken strongly overnight at room temperature. The product was purified by HPLC and analyzed by LC/MS. MW= 1773.3; found [M + H]^+^ = 1774.9.

### Purification of DNA-conjugated compounds

DNA sequences are written in 5’–3’ orientation. 5-Amino modifier C6-CTCTTCCGATCTC on CPG and its HPLC-purified product were ordered from IBA (IBA GmbH, Göttingen, Germany). DNA on controlled pore glass (CPG) was cleaved at 50 °C overnight in 32% NH_4_OH, and the NH_4_OH was evaporated by Speedvac. The DNA was dissolved in aqueous 0.1 M triethylammonium acetate (TEAA buffer, pH 7.0) and then purified on a C18 reverse-phase HPLC column (Phenomenex; Clarity 3 u Oligo-RT) using TEAA buffer/CH3CN gradient on Waters e2695/Waters 2998 systems. Waters ACQUITY UPLC Ultra Performance/Waters ACQUITY TQ Detector equipped with an analytical C18 column (ACQUITY UPLC OST C18, bead size 1.7 μm, 1 × 100 mm; Waters, Milford, Massachusetts, USA) was used to analyze the mass of oligonucleotides. The oligonucleotides were eluted from the C18 column using the gradient of MilliQ ( + 5 mM triethylammonium bicarbonate buffer)/CH3CN( + 5 mM triethylammonium bicarbonate buffer).

### Synthesis of CsA DNA

The CPG beads with the sequence synthesized were washed three times with acetonitrile. The MMT group on the 5’-amino group of oligonucleotide was removed by washing with 3% trichloroacetic acid in DCM until the supernatant was colorless and CPG was washed for an additional three times with DCM to remove trichloroacetic acid. The CPG beads were dried and ready to use. Five microliters of Fmoc-CsA-COOH (200 mM in DMF), 2 μl of HOAt (500 mM in DMF), and 1.9 μl of HATU (500 mM in DMF) were agitated for 1 h. Then, 9.2 μl of DMF was added, and the mixture was centrifuged at 13,000 rpm for 10 min. The supernatant was transferred to a new eppendorf tube and 2 μl of trimethylamine (1.5 M in DMF) was added, then the reaction mixture was added to the CPG beads and shaken overnight at 30 °C. The CsA-conjugated oligonucleotide on the CPG beads were cleaved from CPG, purified, and quantified as described previously. MW= 5275.2 (Fmoc group was removed during the cleavage of CsA DNA from CPG); found [M-3H]^3-^ = 1756.19.

### Synthesis of TAMRA-CsA-DNA

A volume of 100 µL TAMRA NHS (2 mM in dimethyl sulfoxide (DMSO)) was added to 50 µL of CsA-DNA (200 µM in 100 mM TEA-HCl buffer (pH 9.0)), and the mixture was shaken strongly overnight under room temperature. One milliliter of TEAA buffer was added, and the sample was purified and quantified as described previously. MW = 5687.7; found [M-3H]^3-^ = 1894.82.

### Synthesis of SLF-DNA

One hundred microliters of 10 mM SLF-COOH, 15mM N-Hydroxysulfosuccinimide sodium salt (s-NHS), and 10 mM 1-Ethyl-3-(3-dimethylaminopropyl) carbodiimide (EDC) were vortexed for 30 min, and the mixture (SLF-sNHS) was added to 50 µL of 5-amino modifier C6-CTCTTCCGATCTC (200 µM in 100 mM TEA-HCl buffer (pH 9.0)). The mixture was shaken strongly overnight under room temperature. One milliliter of TEAA buffer was added and the sample was purified and quantified as described previously. MW= 4622.2; found [M-3H]^3-^ = 1539.39.

### Synthesis of TAMRA-SLF-DNA

Fmoc-Lys(Mmt)-OH was coupled to 5-amino-modifier-C6-CTCTTCCGATCTC the same way as CsA-DNA. After the purification of Lys(Mmt)-DNA (Fmoc was removed during the oligonucleotide cleavage from CPG), then the TAMRA was coupled to the α amino groups of Lys. After purification of TAMRA-Lys(Mmt)-DNA, the Mmt group was removed by overnight incubation of the DNA with 80% acetic acid. After the purification of TAMRA-Lys-DNA, the SLF was coupled as described before. Mw= 5162.9; found [M-3H]^3-^ = 1719.46.

### Isolation of PBMCs from health and leukemic human blood

Blood and bone marrow samples from healthy donors and patients with AML were obtained after informed consent. The use of banked cells for research purposes within this study had been approved by the local ethics commission of the TU Dresden (EK125042010 and EK206082008).

The blood (10 mL) was diluted in PBS (25 mL) (Thermo Fisher Scientific Inc.) and mixed. The diluted blood was carefully layered over 10 mL of Histopaque®-1077 (Sigma-Aldrich Chemie GmbH). The samples were centrifuged at 600 ×g for 25 min in a swinging-bucket rotor without brake. The upper layer was aspirated and the mononuclear cell layer (lymphocytes, monocytes, and thrombocytes) was carefully transferred to a new 50 mL tube. The tube was filled with PBS, mixed, and centrifuged at 300 × g for 15 min. The supernatant was carefully removed. The pellet was resuspended in 50 mL PBS, mixed, and centrifuged at RT and 300 × g for 10 min. The supernatant was carefully removed completely. The pellet was resuspended in PBS and mixed prior to cell counting and centrifugation at 300 × g for 10 min. PBMCs were proceeded for cryopreservation in freezing medium (heat-inactivated (h.i.) fetal bovine serum (FBS) supplemented with 10% DMSO (v/v)) at a density of 2 × 10^7^ cells/mL.

### Thawing and culture of healthy PBMCs

The cryovial from liquid nitrogen was transferred to a 37 °C water bath with an occasional gentle “flick” for around 1 min. The cell was pooled to a 50 mL polypropylene centrifuge tube containing 8 mL of warm cRPMI (RPMI1640 (Lonza Cologne GmbH, Cologne, Germany) supplemented with 10% (v/v) h.i. FBS (Biochrom GmbH, Berlin, Germany) and 2mM L-glutamine (Life Technologies GmbH, Darmstadt, Germany)). The remaining cells in cryovial were transferred to the 50 mL tube with 1 mL cRPMI. The sample was centrifuged at 250 × g for 7 min. The supernatant was carefully removed. The pellet was resuspended in 5 mL cRPMI and cultured in polystyrene culture flasks (Greiner Bio-One GmbH, Frickenhausen, Germany) for 12–18 h to rest the cells.

### Thawing and culture of leukemia cells

The cryovial was rapidly thawed in a 37 °C water bath. One milliliter of the sample (containing maximal 1 × 10^7^ PBMCs) was transferred to a 15 mL tube and 10 mL of thawing medium (RPMI1640, 5% fetal calf serum, Heparin 20 U/mL, DNAse I 8 U/mL) was added dropwise. The sample was incubated in a water bath at 37 °C for 1 h. Afterwards, the sample was centrifuged at RT and 300 × g for 10 min. The supernatant was carefully removed completely and the pellet was resuspended in 5 mL RPMI1640 + 5% FBS and cultured in polystyrene culture flasks for 12–18 h to rest the cells.

### Cell culture

Human dermal fibroblasts were isolated from neonatal foreskin. Primary human dermal fibroblasts isolated from neonatal foreskin (HDFn) is an adherent cell line purchased from Life Technologies GmbH. HDFn were cultured in Medium 106 (Life Technologies GmbH) supplemented with 1 ×Low Serum Growth Supplement (LSGS) (Life Technologies GmbH) in polystyrene culture flasks (Greiner Bio-One GmbH). For subculturing or prior counting, HDFn were treated with trypsin-EDTA (Life Technologies GmbH) to detach them from the surface. HDFn in suspension were spun down at 1000 rpm for 3 min at room temperature. The pellet was resuspended and transferred to new culture flasks.

*Human mesenchymal stromal cells*: Primary human MSCs were kindly provided by Bornhäuser lab (Department of Internal Medicine I, University Hospital Carl Gustav Carus, Technische Universität Dresden, martin.bornhaeuser@uniklinikum-dresden.de). Adherent MSCs were cultured in Dulbecco's modified Eagle's medium GlutaMax low glucose (1 g/L) (Life Technologies GmbH) supplemented with 10% (v/v) h.i. FBS (Biochrom GmbH) and 2mM L-glutamine (Life Technologies GmbH) in polystyrene culture flasks (Greiner Bio-One GmbH). Growth conditions for all cell lines were maintained in a humidified chamber at 37 °C and 5% CO_2_. For subculturing or prior counting, MSCs were treated with trypsin-EDTA (Life Technologies GmbH) to detach them from the surface. MSCs in suspension were spun down at 1000 rpm for 3 min at room temperature. The pellet was resuspended and transferred to new culture flasks.

*The immortalized leukemia cell lines*: The leukemia cell lines (Jurkat, MOLM-13, K562, HL60, MV4-11, KG-1, and OCI-AML3) were purchased from DSMZ-German Collection of Microorganisms and Cell Cultures (Braunschweig, Germany). Besides OCI-AML3, the other leukemia cells were cultured in cRPMI. OCI-AML3 was cultured in minimum essential medium-α, nucleosides (Thermo Fisher Scientific Inc.) supplemented with 20% h.i. FBS (Biochrom GmbH, Berlin, Germany). Cell lines were maintained at 0.1–2.0 × 10^6^ cells/mL and cultures had to be split every 2–4 days.

### Stiffness measurement by rheometer

Anton appar rheormeter (MCR302) equipped with H-PTD 200 and 20 mm-diameter cone/plate was used to perform the measurements. Continuous flow of dry air was turned on to avoid water condensation at low temperature. The cone and plate were cooled to 4 (for fibrin gel and Matrigel) or 15 °C (for collagen gel), and then the sample was added on to the plate. Once the cone reached the designed gap height, the dry air was shut down and the excess sample was trimmed. To minimize sample evaporation at 37 °C, a tissue paper wet in HEPEs buffer (10 mM, pH 7.8) was placed inside the H-PTD chamber to increase the humidity. To remove the gelled protein on the cone and plate after each measurement, the cone and plate were removed from the machine for cleaning (wiped with tissue paper wet in MilliQ water and rinsed with MilliQ water, then wiped with tissue paper wet in ethanol and rinsed with MilliQ water, then wiped with tissue paper wet in MilliQ water and rinsed with MilliQ water again). After cleaning, the cone and plate were dried with compressed air. The cone and plate were treated with plasma for 5 min to increase the hydrophilicity of the metal surface.

*Matrigel*: Corning™ Matrigel™ Membranmatrix GFR (Corning) was thawed overnight on ice at 4 °C before using. Twenty-five microliters of Matrigel, 20 µL of HEPES/PBS (62.5 mM HEPES buffer in PBS, pH 7.4), 4.5 µL of CypA (100 µM in PBS), and 0.5 µL of CsA (1 mM in 50% MeOH/50% PBS) or 0.5 µL of 50% MeOH/50% PBS were mixed by pipetting in a protein low-binding tube and kept overnight on ice. Forty-five microliters of the mixture was transferred to the rheometer plate for rheology measurement. The measurement was performed with temperature-dependent behavior (a linear increase from 4 to 37 °C in 120 min) oscillations at a strain of 0.1% and frequency of 1 Hz. HEPES buffer was used to maintain the pH because Matrigel stock solution was in Dulbecco's modified Eagle's medium. CsA was dissolved in methanol. DMSO should not be used because it affects Matrigel. For each condition, two independent experiments were performed (Supplementary Fig. 2a).

*Collagen hydrogel*: To 24 µL of the neutralization buffer (28.75 mM NaOH and 62.5 mM HEPES buffer (pH7.4) in 2.25 × PBS), 6 µL of CypA (with desired concentration in 1 × PBS) and 0.3 µL of CsA* (named as [Amino acid-MeBMT] CsA in the previous paper)^21^ (4 mM in DMSO) or 0.3 µL DMSO were added, mixed, and kept on ice for 30 min. Thirty microliters of collagen (5 mg mL^−1^ in 20 mM acetic acid; Type I, rat-tail, Enzo Life Sciences) was added and pipetted up and down ten times. Fifty microliters of the mixture was transferred to the rheometer plate for measurement. This was performed with temperature-dependent behavior (from 4 to 37 °C with a rate of 1 °C per 30 s) oscillations at a strain of 0.1% and frequency of 1 Hz, and was followed by continuous oscillations at 37 °C for 30 min. CsA* was used for its higher solubility in aqueous buffer, when compared to CsA. The pH of the neutralization buffer was 8.5, and after the addition of CypA and collagen, it became 7.4. For each condition, two independent experiments were performed (Fig. 1b and Supplementary Fig. 2b).

*Fibrin gel*: Fibrinogen (Shanghai Laishi Xuezhipin Limited Company) was dissolved in PBS with a concentration of 22.2 mg mL^−1^ overnight at room temperature with gentle shaking and aliquoted in protein low-binding tubes (45 µL per tube). To the tube with fibrinogen, 4 µL of CypA or inactive mutant CypA R55A (a gift from Gunter Fischer) (in PBS) of desired concentrations and 1 µL of CsA (2 mM in DMSO) or 1 µL DMSO were added, mixed by pipetting, and kept on ice for 30 min before measurement. Fifty microliters of thrombin (4 EU mL^−1^ in PBS with 1.8 mM CaCl_2_) was added and pipetted up and down ten times. Then, it was transferred to the rheometer plate for measurement. The plate temperature was raised to 37 °C within 80 s, and the measurement was performed with continuous oscillations at a strain of 0.1% and frequency of 1 Hz for 30 min (30 s per data point). For each condition, two independent experiments were performed (Fig. 1a and Supplementary Fig. 1).

### Viscosity measurement by rheometer

The machine used for viscosity is the same as for stiffness measurements. To 30 µL of 4 mg mL^−1^ fibrinogen, an equal volume of Jurkat cells (8 × 10^7^ mL^−1^ in PBS, washed two times with PBS before used; Jurkat cells ocupated around 20% of the volume) was added. The mixture was pipetted up and down six times, kept on ice for 10 min, and then transferred to the rheometer plater (25 °C) for measurements. The plate temperature was raised to 37 °C. The measurement was performed by increasing shear rate ramp liner from 100 to 1000 s^−1^. Hundred data points were collected during the measurements. For each condition, two independent experiments were performed (Fig. 1c and Supplementary Fig. 3).

### Stability and cellular uptake of DNA-conjugated compounds

A designed concentration of DNA-conjugated compounds (30 µL in PBS) was incubated with 30 µL of PBS or the cell on ice for 10 min, then 20 min at 37 °C, which is much longer then the viscosity assay time. All the samples were centrifuged at 1000 rpm for 3 min, and then, 30 µL of the supernatant was transferred to MS tube for LC/MS analysis (Supplementary Fig. 4).

A final concentration of 100 nM DNA-conjugated TAMRA compounds and Cs9TAMRA was incubated with Jurkat cells for 1 h or 24 h, the cells were pelleted by centrifugation at 1000 rpm for 5 min, the pellet was washed one time with fluorescent-activated cell sorting (FACS) buffer (PBS with 1% bovine serum albumin), and resuspended in FACS buffer. BD FACS Canto™ II Flow Cytometer was used to analyze the uptake of these compounds by Jurkat cells (Supplementary Fig. 5).

### Running method of ultra-performance liquid chromatography

Ultra-performance liquid chromatography (UPLC) (Waters, Milford, USA) was equipped with a widepore C8 column (Aeris™ 3.6 µm WIDEPORE XB-C8 200 Å, LC Column 50 × 2.1 mm) and ACQUITY UPLC PDA Detector. Water with 0.1% formic acid was used as solvent A and acetonitrile with 0.1% formic acid as solvent B. The flow rate was 0.5 mL/min. The wavelength for detection ranged from 300 to 400 nm. The injection volume was 10 µL. The time curve for running was as follows: 0.0–0.5 min, 95% buffer A; 0.5–3.5 min, a linear gradient running from 95% buffer A to 95% buffer B; 3.5–3.6 min, 95% buffer B; 3.6–3.7 min, a linear gradient running from 95% buffer B to 95% buffer A; 3.7–4.0 min, 95% buffer A.

### Substrate proteolytic condition optimization

The assay was performed in HEPES buffer (35 mM HEPES, 150 mM NaCl, 1 mM DTT, pH 7.8). Suc-AFPF-pNA was dissolved in DMSO to a final concentration of 20 mg mL^−1^ and further diluted in HEPES buffer to a final concentration of 600 µg mL^−1^ (ten times the substrate stock). α-Chymotrypsin was prepared in 1 mM HCl with a final concentration of 60 mg mL^−1^. After the termination of proteolysis, the samples were injected to UPLC. The concentration of the substrate and product (pNA) was calculated by the calibration curves (Supplementary Fig. 6).

*Termination condition 1* (Supplementary Fig. 7a,**;** black curve): The assay was performed in Eppendorf tubes on ice, stirring with small stir bars. The assay was started by the addition of 5 µL of α-chymotrypsin to 95 µL of 60 µg mL^−1^ Suc-Ala-Phe-Pro-Phe-pNA in HEPES buffer and stopped by adding 10 µL of 1.1 M HCl at five different time points. HCl caused the precipitation of the protein, and the samples were injected to UPLC after removing the protein by centrifugation.

*Termination conditions 2 and 3* (Supplementary Fig. 7a; red curve and blue curve): Acetic acid was chosen as it is a weak acid, and thus, a high concentration was applied. To have more data points in parallel, the assay was performed in non-binding 96-well plate (Greiner Bio-One GmbH, Kremsmünster, Austria), stirring with small stir bars inside the wells in a cold room. Hundred microliters (termination condition 2) or 200 µL (termination condition 3) of 30% acetic acid was added to 50 µL of the reaction mixtures at different time points (a final concentration of 20 and 24% acetic acid, respectively). The solvent remained clear and was injected to UPLC directly after removing the stir bars. Using 12 stir bars, which could be placed in the wells of a 96-well plate, was inconvenient. To overcome this, the 96-well plate was buried in ice, and the assay was performed as described in Supplementary Fig. 8; the result is shown in Fig. 3.

The substrate/product ratio remained constant in the following 24 h, as analyzed by UPLC (Supplementary Fig. 7d). A time of 120 s was used for reaction termination, as this time point showed the highest sensitivity to various catalytic conditions according to simulations (Supplementary Fig. 7e and f).

### Parallel activity and inhibition measurements

The experiments were carried out in HEPES buffer as shown in Supplementary Fig. 8. For Cyps and FKBP, Suc-AFPF-pNA was used as the substrate and α-chymotrypsin as the auxiliary protease. For Pin1, Suc-AFPF-pNA was used as the substrate and nagarse as the auxiliary protease.

For the inhibition of Cyps, 5 nM CypA, 10 nM CypB, or 50 nM CypD and different concentrations of CsA were tested. For FKPB, 20 nM FKBP and different concentrations of rapamycin were tested. The half maximal inhibitory concentration (IC_50_) was determined by dose–response fitting. The *K*_i_ was calculated according to the equation${\mathrm{K}}_{\mathrm{i}} = {\mathrm{IC}}_{50} - \frac{{[{\mathrm{PPIase}}]}}{2}$^28^, where [PPIase] represents the concentration of PPIase. For each inhibition assay, three independent experiments were performed.

### PPIase activity measurements of CypB immobilized on polymer surface

Ten microliters of His-CypB (2.5 µM, 2 µM, 1.5 µM, and 1 µM) was incubated with 10 µL of settled Ni-NTA macroporous silica (Ni-NTA Spin Kit; Qiagen) for 30 min at 0 °C. Subsequently, 80 µL of HEPES buffer was added and incubated for another 10 min with gentle shaking. The silica was precipitated by gravity for 10 min. Eighty microliters were taken from the supernatant and measured along with the silica fraction (the remaining 20 µL) for PPIase activity. The amounts of CypB in the wells were 25, 20, 15, and 10 pmol. Additionally, the activity of 10 µL of 100 nM His-CypB in solution was measured. Three independent experiments were performed. The result is shown in Supplementary Fig. 9.

### PPIase activity measurements on cell surface

The assay was performed in a cold room. If there was no additional description, the assays were performed in CO_2_-independent medium supplemented with 10% (v/v) h.i. FBS (medium-h.i. FBS) and 2mM L-glutamine. Suc-Ala-Phe-Pro-Phe-pNA was dissolved in DMSO to a final concentration of 20 mg mL^−1^ and subsequenly diluted in medium-h.i. FBS to a final concentration of 120 µg mL^−1^ (two times substrate stock). α-Chymotrypsin was prepared freshly. Fifteen micrograms of α-chymotrypsin was dissolved in 2 mL medium-h.i. FBS, and the pH was adjusted with 10 µL 1 M NaOH. Five millimole CsA, SLF, and CsA/SLF mixture were prepared in DMSO.

### Human dermal fibroblasts and MSC

The cells (10,000 per well) were cultured in 96-well plates for 24 h. The supernatant was removed by turning the plate upside down. The cells were washed three times for 5 min under gentle shaking using supplemented CO_2_-independent medium (100 µL/well), and the supernatant was removed as before. The cells were kept in supplemented CO_2_-independent medium (100 µL/well) for measuring PPIase activity.

The medium was removed with a pipette. Forty microliters of the substrate was added to the wells (a, final concentration of 0.25% DMSO; b, final concentration of 10 µM CsA; c, final concentration of 10 µM SLF; d, final concentration of 10 µM CsA/SLF mixture). The mixture was kept for 5 min without agitation, followed by 1.5 min of agitation (linear shaking by hand with a speed of around 200 rounds/min). Twenty microliters were taken from the supernatant and measured along with the cell fraction (the remaining 20 µL) for PPIase activity. The activity measurements were triggered by adding 20 µL α-chymotrypsin at time 0 min, followed by 1.5 min of agitation, and the reaction was stopped at time 2 min with 80 µL of 20% acetic acid followed by 15 rounds of up and down pipetting mixing. Four independent experiments were performed on the same day. The result is shown in Figs. 3b and 4.

### The suspension cells

One or more flasks of cells (5 mL per flask, depending on the maximum culture density of each cell line) were harvested by centrifuging at 1000 rpm for 3 min. To remove PPIases from the supernatant originating from the culture, cells were washed one time with 2 mL CO_2_-independent medium and were resuspended in 1.6 mL of CO_2_-independent medium with substrate. The cells were centrifuged again, and then 800 µL of the supernatant was taken. The residual volume containing cells was suspended. Nineteen microliters of the cells or supernatant was pipetted to a non-binding 96-well microplate to measure PPIase activity. Four different conditions were performed. Addition of (a) 1 µL medium with 5% DMSO, (b) 1 µL medium with 200 µM CsA, (c) 1 µL medium with 200 µM SLF or (d) 1 µL medium with 200 µM CsA/SLF mixture to the wells. The plate was kept for 5 min without agitation. The activity measurements were triggered by adding 20 µL of α-chymotrypsin at time 0 min, followed by 1.5 min agitation, and the reaction was stopped after 2 min with 80 µL 20% acetic acid, followed by 15 rounds of up and down pipetting mixing. Four independent experiments were performed on the same day. The result is shown in Figs. 3b and 5.

For the Jurkat cell number activity measurements, two flasks of cells (10 mL) were harvested, washed, and suspended in 600 µL of CO_2_-independent medium with substrate. The cells were centrifuged again, and then 300 µL supernatant was taken to measure the activity. The residual volume with cells was suspended and used to measure activity (cell density: 2.4 × 10^7^ cells/mL). Four different conditions were performed: 20 µL supernatant or cell with 0 µL substrate, 10 µL supernatant or cell with 10 µL substrate, 5 µL supernatant or cell with 15 µL substrate, and 2.5 µL supernatant or cell with 17.5 µL substrate. Four independent experiments were performed on the same day. The result is shown in Fig. 6a.

### Reporting summary

Further information on experimental design is available in the Nature Research Reporting Summary linked to this article.

## Supplementary information

Supplementary information

Reporting Summary

Description of Additional Supplementary Files

Supplementary Data 1

Supplementary Data 2

Supplementary Movie 1

## Data Availability

The authors declare that the data supporting the findings of this study are available within the article and its supplementary information files. All source data underlying the graphs and charts presented in the main figures are available in Supplementary Data 1. Leukemia patient characteristics are available in Supplementary Data 2.
