# Supplementary material for: Peptidyl prolyl cis/trans isomerase activity on the cell surface correlates with extracellular matrix development
Source: Commun Biol. 2019 Feb 11;2:58. doi: 10.1038/s42003-019-0315-8 (PMC6370856; doi:10.1038/s42003-019-0315-8)
Supplement: Supplementary file 1 — Supplementary information [file 42003_2019_315_MOESM1_ESM.pdf]

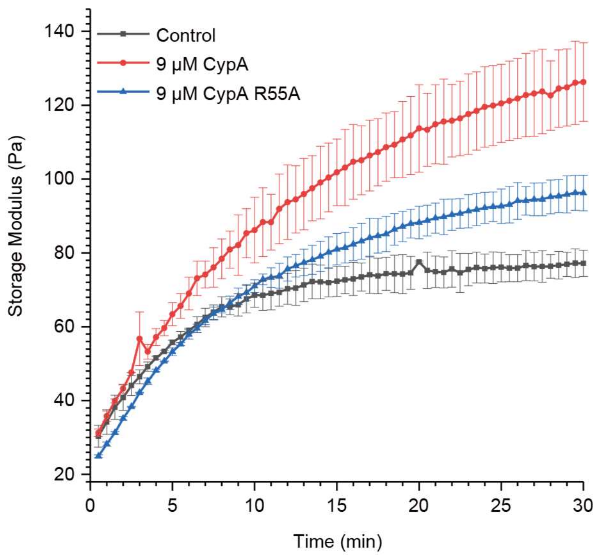

Supplementary Figure 1. Enhanced stiffness (storage modular) of fibrin hydrogel by CypA and its inactive mutant CypA R55A. The effect of CypA mutant on fibrin gelation is remarkably reduced.

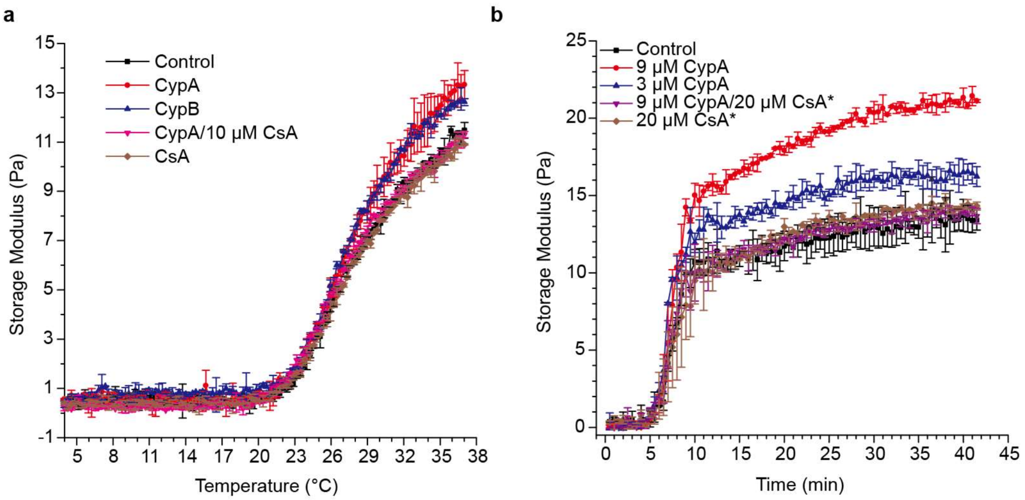

Supplementary Figure 2. Enhanced stiffness (storage modular) of Matrigel (a) and collagen hydrogel (b). The effects can be fully inhibited by cyclophilin inhibitor CsA or CsA*. The gelation of Matrigel was induced by rising the temperature. The gelation of Matrigel was induced by rising the temperature. The gelation of collagen was induced by adjusting the pH to 7.4 on ice followed by rising the temperature. CsA*, a CsA derivative with enhanced solubility.

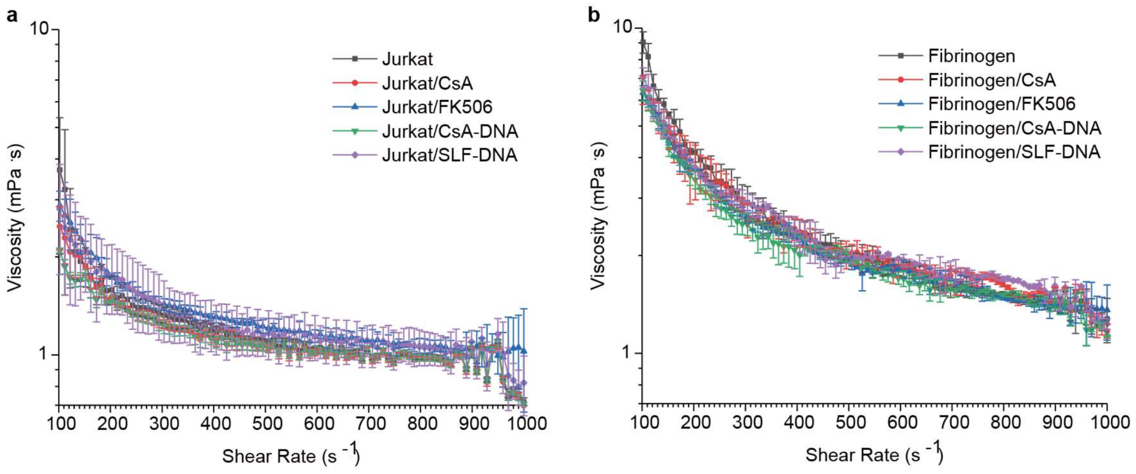

Supplementary Figure 3. Viscosity measurements of Jurkat in PBS (a) and Fibrinogen (b) with or without PPIases inhibitors treatments. PPIases inhibitors has no effect to the viscosity under different shear rate.

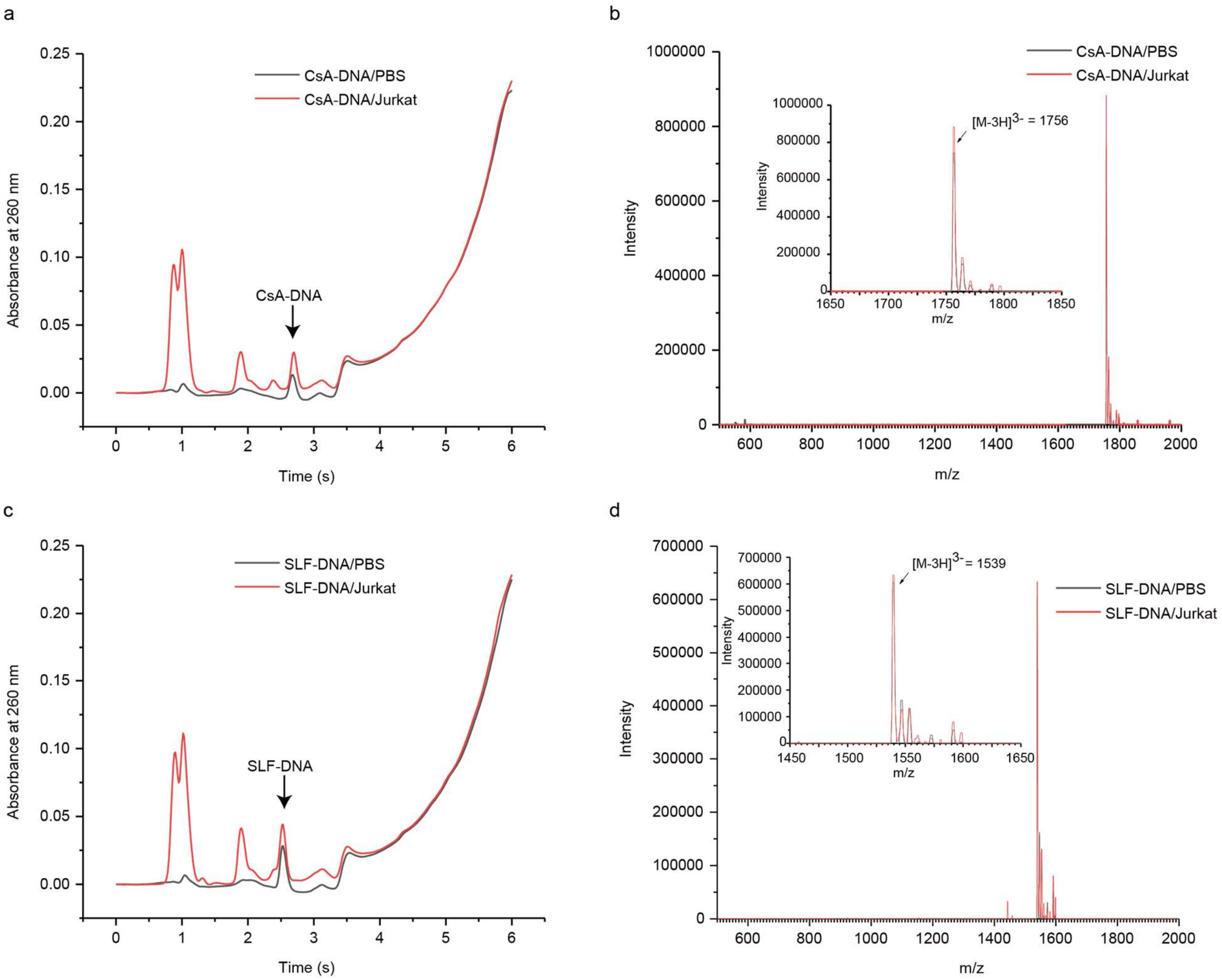

Supplementary Figure 4. (a,c) UV absorbance at 260 nm of CsA-DNA and SLF-DNA. The arrows indicate the elution time of the DNA conjugated compounds and their mass spectrums are shown in b and d. The inset panels are the zoom of mass spectrums. The final concentration of CsA-DNA and SLF-DNA are 500 nM and 1000 nM, respectively. The final concentration of Jurkat cell is 4 X 10^7/mL.

The retention of the DNA conjugated compounds do not change and there no obvious other peaks appear in the mass spectrum after Jurkat cell treatment. These indicate the DNA conjugated compounds are stable under Jurkat cell treatment.

From two independent experiments, the areas of UV are 2218±11, 3054±270, 3626±123 and 4843±58 for CsA-DNA/PBS, CsA-DNA/Jurkat, SLF-DNA/PBS and SLF-DNA/Jurkat, respectively. The Jurkat cell treated DNA conjugated compounds has higher intensity then PBS control, from both the UV absorbance and mass spectrum. This can be due to the Jurkat cell also occupied some volume. These also indicated DNA conjugated compounds do not diffuse inside the cell.

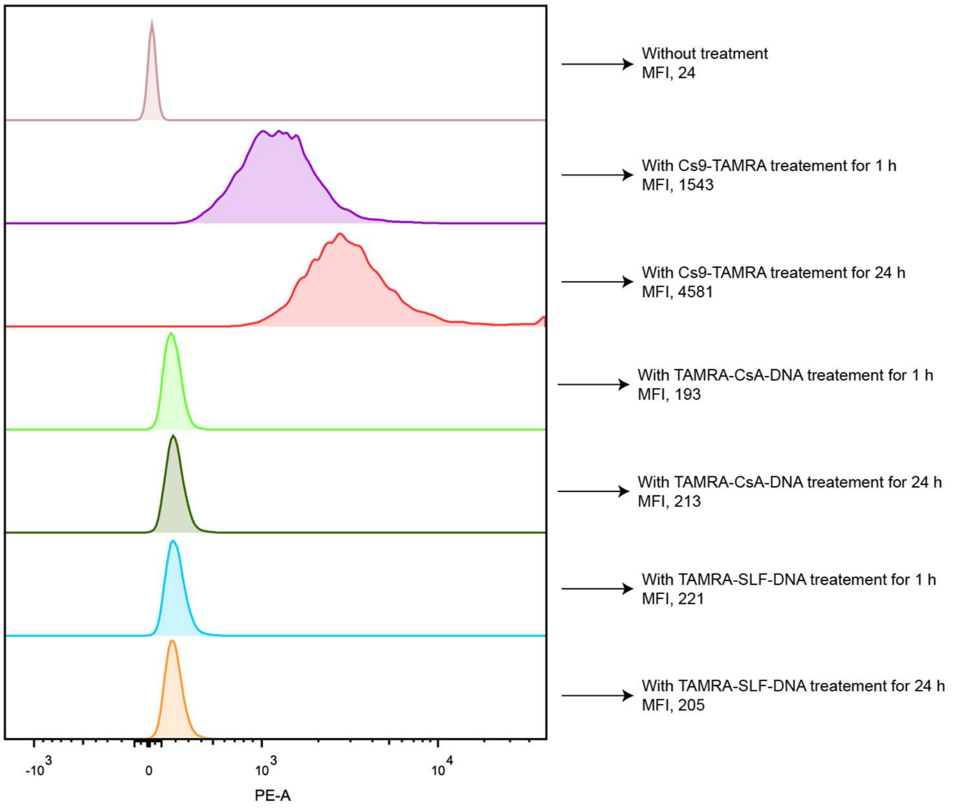

Supplementary Figure 5. Histograms of Jurkat cell fluorescence after incubation with Cs9-TAMRA, TAMRA-CsA-DNA and TAMRA-SLF-DNA for 1 and 24 hours, or reference cells without treatement. For the samples with the treatment of DNA conjugated compounds, the fluorescence intensity is much lower than that Cs9-TAMRA and do not behaviors as time dependent uptake. These indicated DNA conjugated compounds do not enter the cell. MFI, mean fluorescence intensity.

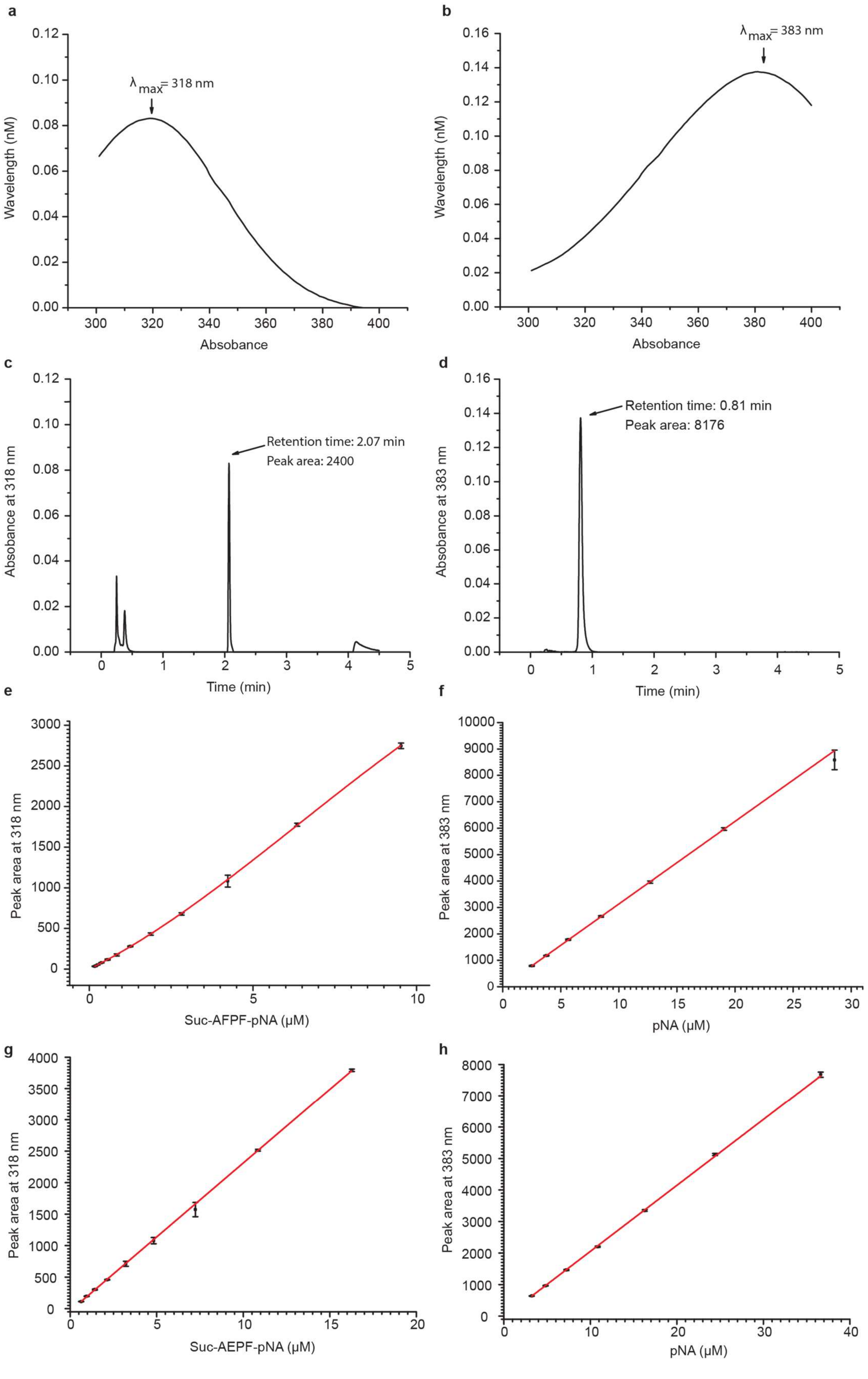
a
λmax= 318 nm
Wavelength (nM)
Absobance
b
λmax= 383 nm
Wavelength (nM)
Absobance
c
Retention time: 2.07 min
Peak area: 2400
Absobance at 318 nm
Time (min)
d
Retention time: 0.81 min
Peak area: 8176
Absobance at 383 nm
Time (min)
e
Peak area at 318 nm
Suc-AFPF-pNA (μM)
f
Peak area at 383 nm
pNA (μM)
g
Peak area at 318 nm
Suc-AEPF-pNA (μM)
h
Peak area at 383 nm
pNA (μM)

Supplementary Figure 6. Calibration curves of Suc-AFPF-pNA, Suc-AEPF-pNA and pNA by UPLC.
(a-d) The UV spectra and retention times of Suc-AFPF-pNA (a,c) and pNA(b,d). (e,f) Calibration curves of Suc-AFPF-pNA ($y = 1.10 + 198.11x + 18.87x^2 – 0.98x^3$, (e)) and pNA ($y = 4.56 + 312.67x$, (f)). pNA was obtained by treating Suc-AFPF-pNA with 3 mg/ml α-chymotrypsin from bovine pancreas for 5 min at room temperature. (g,h) Calibration curves of Suc-AEPF-pNA ($y = -35.24 + 234.72x$, (g)) and pNA ($y = -39.55 + 209.40x$, (h)). pNA was obtained by treating Suc-AEPF-pNA with 3 mg/ml Protease Type XXIV (nagarse , Sigma-Aldrich GmbH, St. Louis, MO, USA) for 5 min at room temperature.The peak areas were integrated by Masslynx software at 318 nm for Suc-AFPF-pNA (or Suc-AEPF-pNA) and 383 nm for pNA, respectively. Three independent experimental data were plotted using different concentrations of compound (X axis) against correlated peak area (Y).

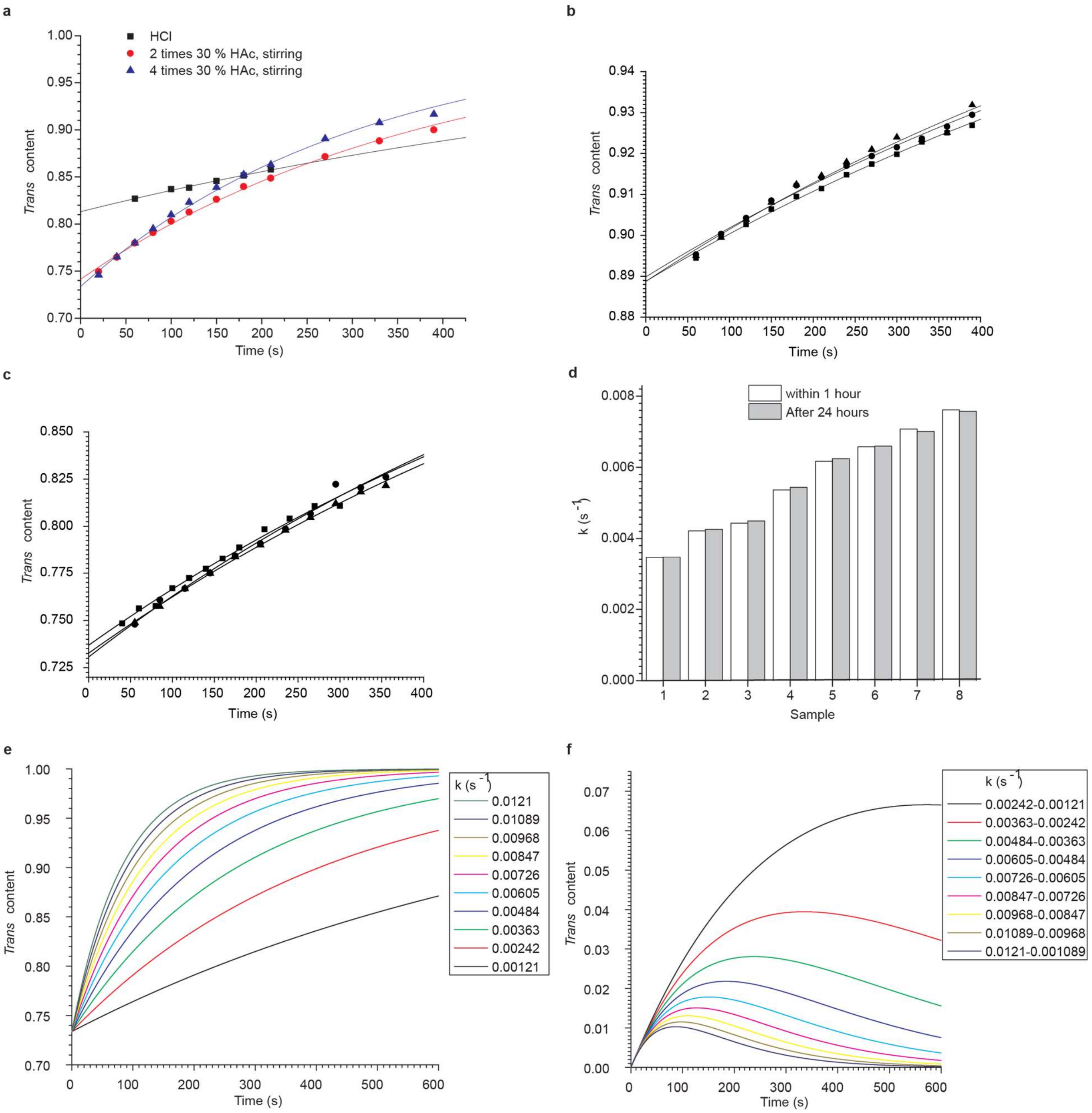

Supplementary Figure 7. Assay optimization. (a) termination of α-chymotrypsin hydrolysed Suc-AFPF-pNA by adding acids under stirring. Using HCl (black curve), the initial *cis* content (from fitting data) was 18.7 ± 0.1% and the spontaneous isomerization rate of peptide was 1.29 ± 0.05 × 10^{-3} s^{-1}. The lower value of initial cis content (only 18.7 ± 0.1% compared to 27 ± 2% by NMR[23]) was because HCl could not stop the proteolysis instantly and the α-chymotrypsin kept hydrolyzing the peptide. Increasing the final HCl concentration may solve this problem but could be harmful to the column equipped on UPLC. Using acetic acid, the initial *cis* content was 25.7 ± 0.2% for final 20 % acetic acid (red curve) and 26.6 ± 0.2% for final 24 % acetic acid (blue curve), which was in good agreement with the data published. Both conditions could stop the hydrolysis instantly. The spontaneous isomerization of peptide itself was different (2.57 ± 0.05 × 10^{-3} s^{-1} for red curve, 3.23 ± 0.07 × 10^{-3} s^{-1} for blue curve), which may be due to the unstable temperature in cold room. The data was fitted with first order according to Fig. S3. (b, c) Termination of α-chymotrypsin hydrolysed Suc-AFPF-pNA (b) and nagarse hydrolysed Suc-AEPF-pNA (c) by adding acetic acid at different time points. The initial *cis* content of Suc-AFPF-pNA was 26.63 ± 0.31% and the spontaneous isomerization rate of peptide was 1.21 ± 0.05 × 10^{-3} s^{-1}. The initial *cis* content of Suc-AEPF-pNA was 11.09 ± 0.06% and the spontaneous isomerization rate of peptide was 1.16 ± 0.06 × 10^{-3} s^{-1}. The data was fitted with first order according to Fig. S2, using origin software. (d) Stability of Suc-AFPF-pNA and pNA after inactivation of α-chymotrypsin. The isomerization rates of 8 different terminated reaction mixtures calculated from the injections within 1 hour (white bars) and after 24 hours (grey bars). No difference of calculated isomerization rates between the injections within 1 hour and after 24 hours. (e, f) Simulation curves of α-chymotrypsin mediated proteolysis of Suc-AFPF-pNA.(E) First order hydrolysis curves of Suc-AFPF-pNA with different isomerization rates as indicated in the inserted rectangle. Simulation curves were prepared with the assumption that the maximum *cis* to *trans* isomerization rate was 0.012 s^{-1} (10 times of spontaneous isomerization

rate) upon enzyme catalysis and that enzyme activity was inhibited to various degrees. (f) Discrimination of neighboring curves from panel A. Data was obtained as indicated in the inserted rectangle. For example, the black curve of panel b represents the subtraction value of red curve (panel A, k = 0.00242 s^{-1}) by black curve (panel A, k = 0.00121 s^{-1}). Obviously, too short as well as too long times resulted in low discrimination for most of the curves. The best discrimination was achieved from 80 s to 200 s. 120 s was chosen as time for reaction termination in the following experiments.

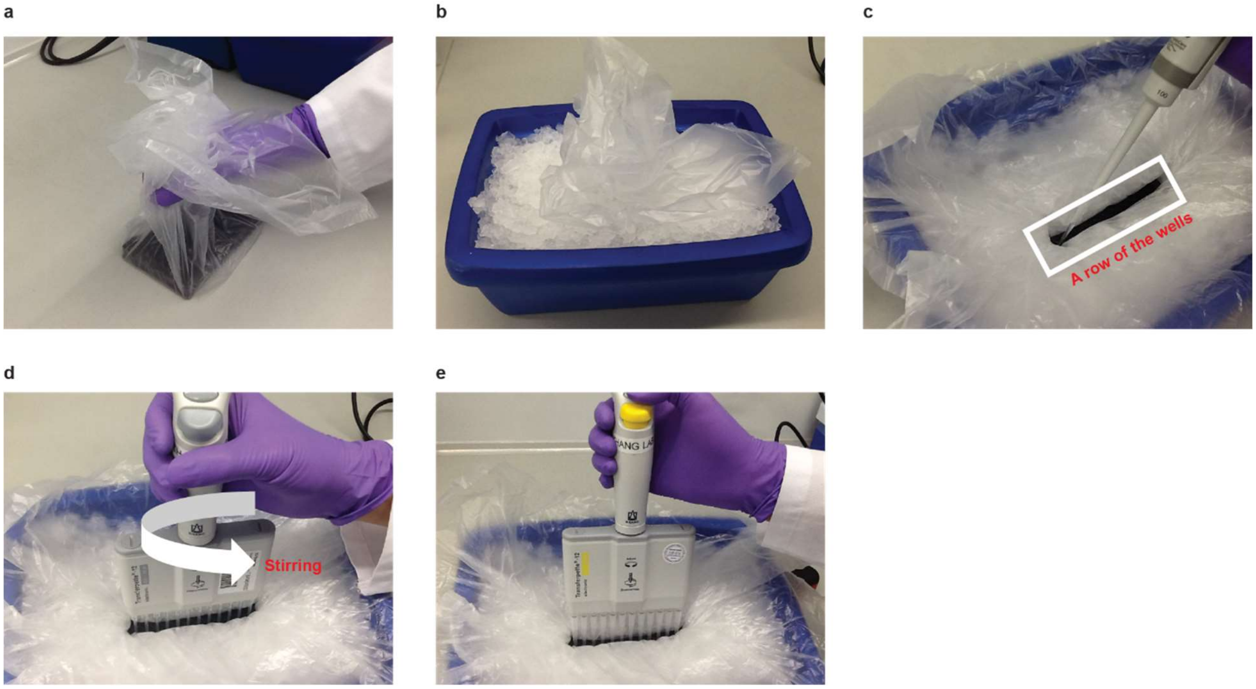

Supplementary Figure 8. Peptidyl prolyl isomerase activity measurement procedure. (a-c) A non-binding 96 well microplate was wrapped with a plastic film (a) and buried in ice (b) for 10 min to reach equilibration temperature. A small gap was opened on the top of a row, and then different reaction mixtures (95 µL of 60 µg/mL substrate in HEPES buffer with/without PPIase and various inhibitor concentrations; Suc-AFPF-pNA for Cyps and FKBP12, Suc-AEPF-pNA for Pin1) were added to the different wells separately in the same row and incubated for 20 min (c). (d)To trigger the reaction, 5 µl of 60 mg/ml proteinase (α-chymotrypsin for Cyps and FKBP, nagarse for Pin1) was added to each well simultaneously by multi-channel pipet and the reaction mixtures were stirred quickly with the tips, in one direction, for 30 seconds.
(e)To stop the reactions 120 seconds later, 200 µl of 30% acetic acid were added to the wells and the reaction mixtures were mixed by pipetting up and down 15 times using the multi-channel pipet. After stopping the reactions with 30 % acetic acid, the mixtures were injected on an UPLC and the ratios between substrate and pNA were calculated according to the calibration curves.

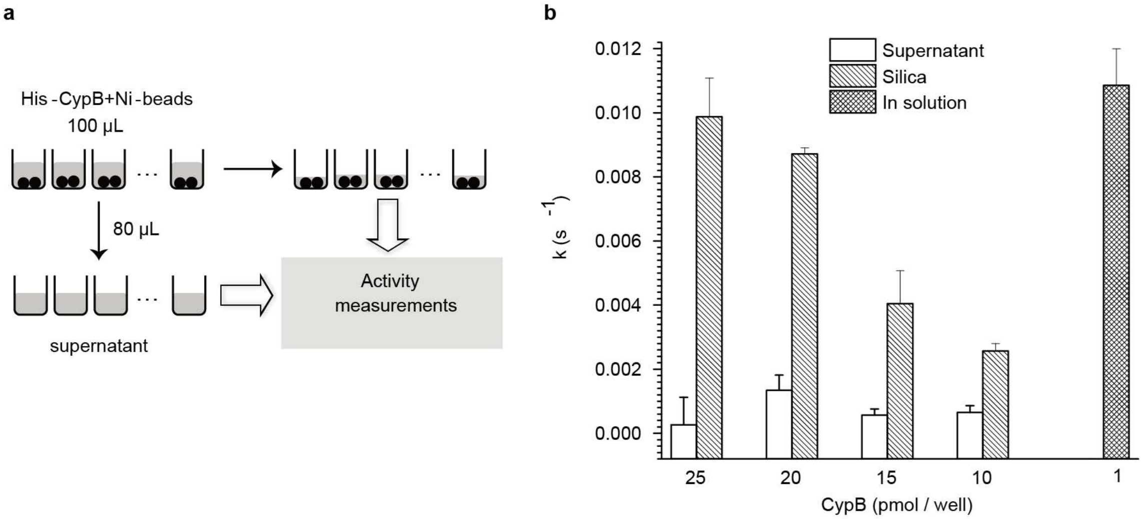

Supplementary Figure 9. Activity measurement of His-CypB immobilized on Ni-silica and in solution. (a) Scheme of the assay procedure. Different amounts of CypB were immobilized on Ni-NTA macroporous silica. Both the supernatant and silica fractions were taken to measure the PPIase activity. (b) Activity measurements of His-CypB on the silica fractions, in supernatant fractions and in solution. The activity of His-CypB on the silica fractions resulted mainly from immobilized His-CypB since the supernatant showed no activity. The control of 1 pmol free His-CypB in solution revealed a diminished catalytic activity of immobilized His-CypB, as 25 pmol only showed a similar activity to the control.

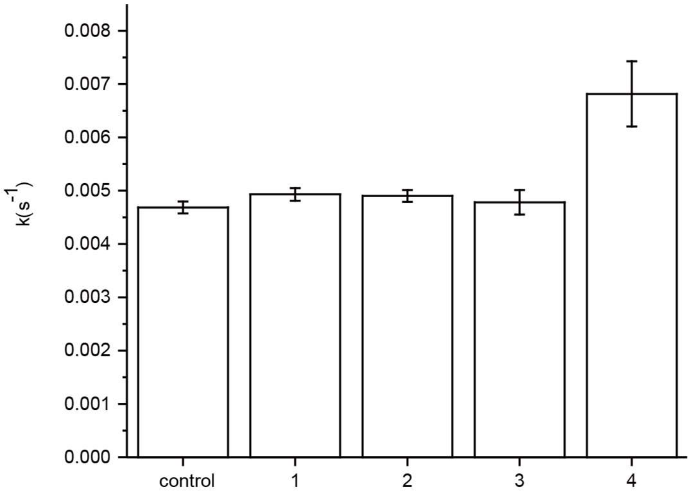

Supplementary Figure 10. Multiple times washing of Jurkat by centrifugation. There are no different between 1 to 3 times and the medium control. One time washing is enough to exclude contamination from PPIases in the medium.

Supplementary Table 1. Enzyme inhibition of DNA conjugated compounds (final concentration 100 nM).

Sample	Relative enzyme activity (Enzyme only as 100%)
**10 nM CypA/ 100 nM CsA-DNA**	4.02194 ± 0.25373%
**10 nM CypA/ 100 nM TAMRA-CsA-DNA**	2.89871 ± 1.81232%
**30 nM FKBP/ 100 nM SLF-DNA**	2.69305 ± 0.32742%
**30 nM FKBP/ 100 nM TAMRA-SLF-DNA**	2.21179 ± 0.03563%

Supplementary Table 2. The sequences of recombinant proteins

CypA	MKHHHHHHMKQVNPTVFFDIAVDGEPLGRVSFELFADKVPKTAENFRALSTGEKGFGYKGSCFH RIIPGFMCQGGDFTRHNGTGGKSIYGEKFEDENFILKHTGPGILSMANAGPNTNGSQFFICTAK TEWLDGKHVVFGKVKEGMNIVEAMERFGSRNGKTSKKITIADCGQLE
CypB	MKHHHHHHMKQLLPGPSAADEKKKGPKVTVKVYFDLRIGDEDVGRVIFGLFGKTVPKTVDNFVA LATGEKGFGYKNSKFHRVIKDFMIQGGDFTRGDGTGGKSIYGERFPDENFKLKHYGPGWVSMAN AGKDTNGSQFFITTVKTAWLDGKHVVFGKVLEGMEVVRKVESTKTDSRDKPLKDVIIADCGKIE VEKPFAIAKE
Cyp40	MKHHHHHHMKQSHPSPQAKPSNPSNPRVFFDVDIGGERVGRIVLELFADIVPKTAENFRALCTG EKGIGHTTGKPLHFKGCPFHRIIKKFMIQGGDFSNQNGTGGESIYGEKFEDENFHYKHDREGLL SMANAGRNTNGSQFFITTVPTPHLDGKHVVFGQVIKGIGVARILENVEVKGEKPAKLCVIAECG ELKEGDDGGIFPKDGSGDSHPDFPEDADIDLKDVDKILLITEDLKNIGNTFFKSQNWEMAIKKY AEVLRYVDSSKAVIETADRAKLQPIALSCVLNIGACKLKMSNWQGAIDSCLEALELDPSNTKAL YRRAQGWQGLKEYDQALADLKKAQGIAPEDKAIQAELLKVKQKIKAQKDKEKAVYAKMFA
Pin1	MKHHHHHHMKQADEEKLPPGWEKRMSRSSGRVYYFNHITNASQWERPSGNSSSGGKNGQGEPAR VRCSHLLVKHSQSRRPSSWRQEKITRTKEEALELINGYIQKIKSGEEDFESLASQFSDCSSAKA RGDLGAFSRGQMQKPFEDASFALRTGEMSGPVFTDSGIHIILRTE
