# Supplementary material for: Peptidyl prolyl cis/trans isomerase activity on the cell surface correlates with extracellular matrix development
Source: Commun Biol. 2019 Feb 11;2:58. doi: 10.1038/s42003-019-0315-8 (PMC6370856; doi:10.1038/s42003-019-0315-8)
Supplement: Supplementary file 3 — Description of Additional Supplementary Files [file 42003_2019_315_MOESM3_ESM.docx]

**Description of Additional Supplementary Files**

**File Name**: Supplementary Data 1

**Description**: Source data of the main figures.

**File Name**: Supplementary Data 2

**Description**: Leukemia patient characteristics.

**File Name**: Supplementary Movie 1

**Description**:Video abstract for the paper.
